# IVIG and under Burn Unit Care Yield Favorable Outcomes in Pediatric Patients with Toxic Epidermal Necrolysis: A Case Report and Literature Review

**DOI:** 10.1155/2020/6274053

**Published:** 2020-01-30

**Authors:** Tareq Z. Alzughayyar, Wasim Noureddin Ibrahim Hamad, Eman A. S. Abuqweider, Bilal Nabeel Mohammad Alqam, Sadi A. Abukhalaf, Rami A. Misk, Fawzy M. Abunejma, Jihad Samer Zalloum, Mohanad Saleh, Ali A. Abumunshar, Yousef I. M. Zatari

**Affiliations:** ^1^Al-Quds University, Faculty of Medicine, Jerusalem, State of Palestine; ^2^An-Najah National University, Faculty of Medicine & Health Sciences, Nablus, State of Palestine; ^3^Al-Ahli Hospital, Hebron, State of Palestine; ^4^Intensive Care Unit Al-Ahli Hospital, Hebron University, Hebron, State of Palestine

## Abstract

Body reactions to drugs can manifest as Stevens–Johnson syndrome (SJS) and toxic epidermal necrolysis (TEN). TEN is the most severe form of cutaneous reactions with an incidence rate of 1-2 per million cases per year. Despite TEN being a critical and life-threatening condition, there is little to no evidence of clear management protocol. We reported a 5-year-old male child who presented with lamotrigine-induced TEN and was successfully treated with intravenous immune globulin (IVIG) with a burn unit care level, while TEN treatment with IVIG is an appropriate approach with predictable good outcomes, burn unit care is also effective in creating highly favorable effects. Upon reviewing the literature, several studies indicate that TEN patients treated with the combination of IVIG and burn unit care lead to decreased levels of morbidity and mortality than when treated with IVIG or burn unit care alone. Therefore, treatment involving both IVIG and burn unit care should be considered for TEN patients.

## 1. Introduction

Toxic epidermal necrolysis (TEN) is a severe cutaneous reaction to drugs or their metabolites with multisystem involvement. With a high mortality rate that is greater than 30% [1], low annual incidence of 1-2 cases per million people [2, 3], and largely unknown pathogenesis—TEN is identified as a cutaneous hypersensitivity reaction and is regarded as the most severe of its type, capable of affecting more than 30% of the total body surface area (TBSA). It is advocated that TEN patients are treated in major burn centers that oversee specialized care in protecting vital organs, dressing care, and infection prevention during the process of re-epithelialization [4]. Furthermore, the largest trial to date showed a decreased mortality rate from 51.4% to 29.8% after transfer to a burn unit within 7 days [5].

Despite the significant acute and chronic morbidity associated with the disease, recent advances of treatment protocols are limited in regard to quantity and adequate efficacy. Optimal therapies for patients with TEN remain unclear. Primarily, the treatment of TEN poses itself as a challenge due to the rarity of the condition and the limited number of studies that discuss alternative or varying options for TEN treatment.

In our paper, our suggested method of approach is distinguished by the application of both burn care and IVIG, which would be working in tandem on the premises of combatting and treating TEN. In response to our TEN-patient case, this course of action proved to be highly effective. While reviewing the literature, we also found that this method decreases mortality in other cases that used the same approach.

## 2. Case Presentation

**Figure 1:**
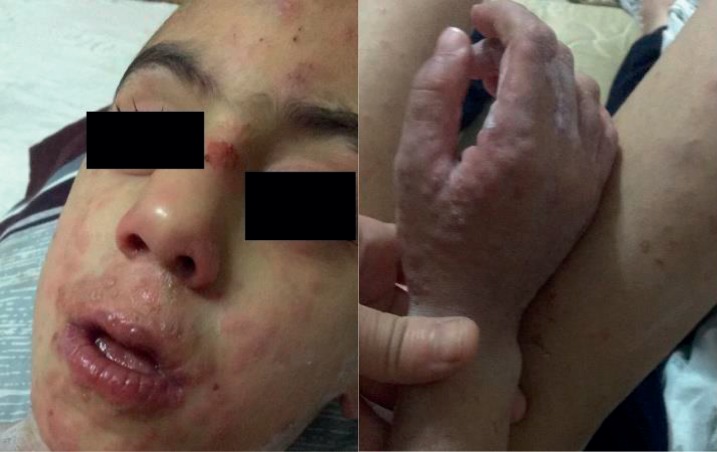
The vesicular lesion involves the hand and mouth of the patient.

A 5-year-old male with one week of decreased activity was admitted due to fever and diffuse rash. A known case of Fragile X syndrome was in his usual state of health until the age of 3 years when he had drop attacks which were managed with valproic acid prescribed by a neurologist. Two weeks before admission, lamotrigine was added to control his symptoms. Two days before admission, the patient developed fever and papular skin rash on his hands, feet, and oral cavity which was treated by an external doctor as hand-foot-mouth disease (Figure 1). On the day of admission, he was brought to the hospital due to worsening in his symptoms with difficulty in feeding. As soon as he arrived, lamotrigine was stopped.

**Figure 2:**
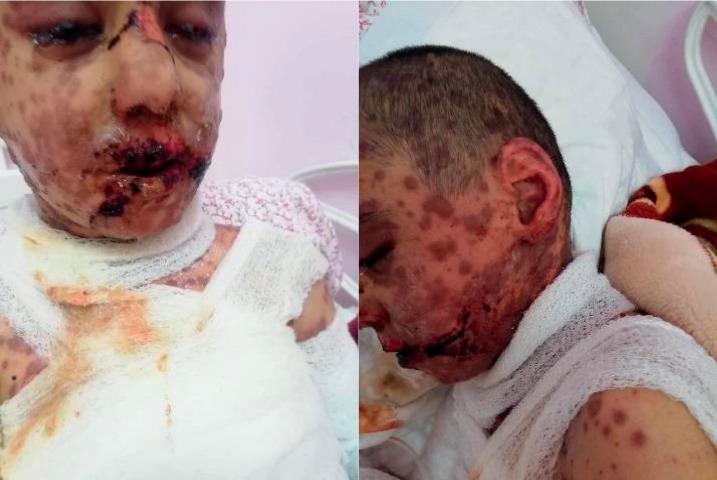
Lesions involving the whole head and face including eyes.

On examination, the patient looked ill and toxic. His temperature, heart rate, blood pressure were, respectively, 39°C, 125 bpm, and 117/81, and his weight was 18 kg. He had extensive bullous skin lesions on his face, hands, feet, oropharynx, and trunk along with hemorrhagic crusting of the lips and bilateral oral conjunctivitis and presented with Nikolsky sign positive (Figure 2). No genital and perianal blisters were found. No splenomegaly, hepatomegaly, and lymphadenopathy were observed.

Laboratory examination results were WBC 6.8 cells/mm, and the differential count was normal. Platelet 180, hemoglobin 12.4, SGPT 18, SGOT 46, creatinine 0.6, BUN 14, NA+ 142, CRP 36, ESR 9, and both INR and aPTT were normal, and SCORTEN score was calculated (Table 1). Eye swap showed *Staphylococcus aureus* growth, and skin biopsy revealed full-thickness epidermal necrosis.

**Figure 3:**
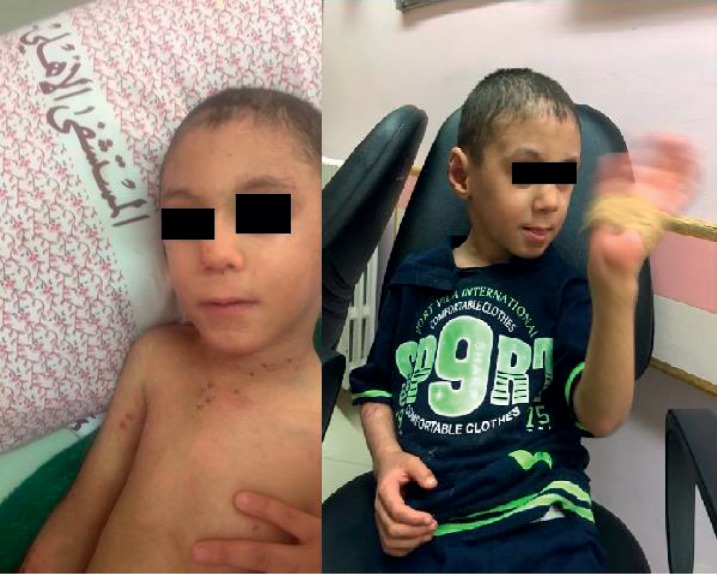
Lesion resolves completely after treatment.

Due to the relatively normal workup and previous medication history, toxic epidermal necrolysis was considered. Due to the large area of involvement (more than 50%), the patient was managed in the burn unit by a multidisciplinary team with strict isolation including isolated room and barrier isolations (gloves and gown), daily wound dressing with local Bactroban application, IV ceftriaxone stopped after negative blood culture, and good hydration (3 times the maintenance; maintenance was 1400 ml/day). Besides that, IVIG (1 mg/kg/day) for 3 days—unfortunately, IVIG is very expensive (3000 USD daily) and not covered by governmental health insurance—and methylprednisone (1.5 mg/kg/day) for 3 days were given. Eye lubrication and antibiotics were considered, and IV paracetamol was used to manage his pain. On the 4th day, improvement was seen by the peeling of skin over his hands, feet, and trunk. His lab workup was normal, and CRP was 26. Upon discharge on the 8th day, the patient was active, his skin was completely recovered (Figure 3), and CRP was 3.

## 3. Discussion

TEN is a rare mucocutaneous hypersensitivity reaction involving greater than 30% of the total body surface area (TBSA) and is most commonly triggered by medication. Even though it is a rare disorder, it is considered a significant concern due to its high mortality rate that reaches 30% [6]. Since its major cause of death is sepsis [7] followed by acute respiratory distress, cardiac arrest, and renal failure, early diagnosis and management are very critical.

TEN is a systemic disease that affects multiple organs. Hence, its management requires that of a multidisciplinary team composed of physicians for both wound and medical management. Although there is no evidence-based protocol for the management of TEN, many studies are acknowledging and affirming the survival benefits for TEN patients when treated in a burn unit [8–10] despite the variation among burn centers in relation to their systemic interventions applied to TEN patients. It is also possible that some survivors of TEN may develop severe, chronic, and often debilitating complications that can permanently impair activities of daily living and quality of life [11].

In a comprehensive compilation and analysis of the literature and data gathered from multiple TEN-focused studies, Table 2 reviews 39 reported cases for TEN patients who are less than 18 years. From these 39 patients, 29 patients were treated in a burn unit and had a mortality rate of 10.3%, whereas the 17 patients that were treated with IVIG had a mortality rate of 5.8%. Based on the literature, the application of both approaches in this age group may cause improvement in mortality. Unfortunately, limited data due to the rarity of the disease prevent us from proving this result (Table 2).

In addition to stopping culpirt trigger(lamotrigine in our case), the main principles of supportive care are the same as major burns and include wound care, fluid and electrolyte management, nutritional support, temperature management, pain control, and monitoring and treating superinfections [19, 20]. Several studies indicate a better prognosis for patients transferred promptly to a burn care unit or intensive care unit. In a retrospective multicenter review of 199 TEN patients treated at burn care centers, the overall mortality was 32% as compared with 51% among patients initially cared for in other settings but were then transferred to a burn center more than one week after disease onset [21].

According to the literature, a high dose of IVIG (2 gm\kg) improved the efficacy of patient recovery as opposed to the patients who did not receive it [8, 9]. Furthermore, a recent meta-analysis of IVIG use in SJS\TEN showed decreased mortality amongst pediatric patients [9]. Studies supporting the rapid recovery after administration of IVIG suggest that the pathophysiology of TEN is related to the abnormal interaction between the cell-surface death receptor Fas and its respective ligand (FasL), which leads to keratinocyte apoptosis and detachment. IVIG has immunomodulatory properties by causing FcR blockade as well as providing anti-idiotypic antibodies to auto-antibodies. Despite there not being a standard management protocol in SJS\TEN, IVIG is still considered a key treatment based on the supporting evidence of many studies that report beneficial outcomes and reduced mortality of using IVIG in SJS\TEN patients [17, 22–25].

Furthermore, corticosteroid was used in the reported patient even though the benefit is controversial. Some studies report an increase in the risk of infection and a higher mortality rate with steroid administration [10, 26]. However, other studies advocate using short-term yet high-dose corticosteroids for drug hypersensitivity reactions [19]. In addition, two retrospective studies suggested that using steroids at an early stage of the disease might be beneficial in decreasing the mortality rate without affecting healing time [23, 24].

## 4. Conclusion

Treatment of TEN is still a challenge due to rarity of disease and limited number of studies about the best treatment approach, and we suggest a new approach in patients less than 18 years which includes IVIG and burn care as mentioned in the case presentation to be considered in the future case-control study because it shows decrease in mortality by reviewing the literature of the previous case report and series which use the same approaches in this age group.

## Figures and Tables

**Table 1:** SCORTEN score 3.

Prognostic factor	Value in index case	Score
Age	5	0
Malignancy	No	0
Heart rate	125	1
BUN	14	0
BSA	50%	1
HCO_3_	18	1
Blood glucose	88	0

**Table 2:** Literature review of 39 cases of TEN (treatment approaches and mortality rate).

Study	Number of patients	TEN : SJS	M : F	IVIG (*n*)	IVCS (*n*)	Another drug modality	TBSA% (mean ± SD)	BU/ICU (*n*)	Mortality (*n*)
Adzick et al. [12]	4	4 : 0	2 : 2	0	0	—	86.25 ± 10.23	4	1
Inamo et al. [13]	3	2 : 1	3 : 0	0	0	Ulinastatin	N/A	0	0
Zahra and Soror [14]	1	1 : 0	0 : 1	1	0	—	90%	1	0
Moudgil et al. [15]	2	0 : 2	1 : 1	2	0	—	N/A	0	0
Prais et al. [16]	4	N/A	0 : 4	4	4	—	N/A	0	0
Spies et al. [11]	15	15 : 0	9 : 6	0	0	—	76 ± 5	15	1
Stella et al. [17]	9	9 : 0	4 : 5	9	9	LMWH	61	9	1
Straussberg et al. [18]	1	0 : 1	0 : 1	1	1	—	N/A	0	0

TEN: toxic epidermal necrolysis, SJS: Stevens–Johnson syndrome, IVIG: intravenous immunoglobulin, IVCS: intravenous corticosteroid, TBSA: total body surface area affected, BU: burn unit, ICU: intensive care unit, LMWH: low molecular weight heparin, and N/A: not available.
